# Pleomorphic linkers as ubiquitous structural organizers of vesicles in axons

**DOI:** 10.1371/journal.pone.0197886

**Published:** 2018-06-04

**Authors:** Nikolas Schrod, Dimitri Vanhecke, Ulrike Laugks, Valentin Stein, Yoshiyuki Fukuda, Miroslava Schaffer, Wolfgang Baumeister, Vladan Lucic

**Affiliations:** 1 Max Planck Institute of Biochemistry, Am Klopferspitz 18, Martinsried, Germany; 2 Institute of Physiology II, University of Bonn, Bonn, Germany; University of Edinburgh, UNITED KINGDOM

## Abstract

Many cellular processes depend on a precise structural organization of molecular components. Here, we established that neurons grown in culture provide a suitable system for in situ structural investigations of cellular structures by cryo-electron tomography, a method that allows high resolution, three-dimensional imaging of fully hydrated, vitrified cellular samples. A higher level of detail of cellular components present in our images allowed us to quantitatively characterize presynaptic and cytoskeletal organization, as well as structures involved in axonal transport and endocytosis. In this way we provide a structural framework into which information from other methods need to fit. Importantly, we show that short pleomorphic linkers (tethers and connectors) extensively interconnect different types of spherical vesicles and other lipid membranes in neurons imaged in a close-to-native state. These linkers likely serve to organize and precisely position vesicles involved in endocytosis, axonal transport and synaptic release. Hence, structural interactions via short linkers may serve as ubiquitous vesicle organizers in neuronal cells.

## Introduction

The precise spatial organization of molecular complexes is essential for many cellular processes. This is especially true for neurons, where large distances and tight spatio-temporal control at synapses require specific, carefully orchestrated localization.

Long-distance, microtubule-based axonal transport is known to involve two distinct organelles. Piccolo-bassoon transport vesicles (PTV) carry active zone scaffolding proteins [1–4], while synaptic vesicle proteins are transported via synaptic vesicle protein transport vesicles (STV) [5, 6]. The vesicular organization of transport packages however has not been clarified. While 80 nm dense core vesicles (DCVs) are constituents of PTVs, and STVs are thought to be composed of clear lumen vesicles, PTVs and STVs may be co-transported [7–9]. Related, clusters of synaptic vesicles and transport packages can accumulate in the absence of postsynaptic terminals [10, 11]. Such nonsynaptic axonal boutons, described as isolated axons, orphan synapses, or mobile synaptic vesicle pools, were reported to be capable of transmitter release [12–16].

Short filaments interlinking synaptic vesicles were observed by electron microscopy (EM) in preparations that involved rapid freezing and sample dehydration [17–21]. Subsequent cryo-electron tomography (cryo-ET) work on synaptosomes identified filaments that interconnect synaptic vesicles (connectors) and tether the vesicles to the active zone membrane as the most prominent structural organizers of the presynaptic terminal [22]. Automated filament detection and analysis showed that these are dynamic structures that respond to synaptic stimulation and are involved in synaptic vesicle release. Similar filaments were also observed in tomograms of high pressure frozen and dehydrated hippocampal slice cultures and dissociated cultures [21, 23].

EM preparation methods like chemical fixation, dehydration and heavy-metal staining have been essential for our current understanding of the neuronal ultrastructure. However, because these methods are known to induce membrane deformation, rearrangements and aggregation of cytosolic material, even when the initial aldehyde fixation is replaced by high pressure freezing to obtain better preservation, these methods obscure fine biological structures and preclude interpretation at the molecular level [24–28]. In Cryo-ET, samples are first rapidly frozen (vitrified), which prevents water crystallization and a rearrangement of the biological material, and are imaged by transmission EM in the same vitrified, fully hydrated state [29]. Therefore, cryo-ET is uniquely suited for high resolution, direct three-dimensional (3D) imaging of cellular complexes together with their environment in the native, unperturbed state [30]

Here we imaged neurites of rodent hippocampal neurons grown in culture by cryo-ET. Our data extends previous findings about the endocytosis, cytoskeletal organization and the morphology of transport processes and their components. Importantly, we found that medium sized transport and endosomal intralumenal vesicles are linked via short, pleomorphic filaments. Because we also confirmed that similar filaments link synaptic vesicles, our data argues that these filaments are ubiquitous organizers in neuronal cells.

## Materials and methods

### Neuronal cultures

Rat hippocampal neurons were prepared for cryo-ET as previously reported [31, 32]. Briefly, gold finder EM-grids (type NH2A by Plano, Wetzlar, Germany) coated with Quantifoil R2/2 or R1/4 (Quantifoil Micro Tools, Jena, Germany) were sterilized in ethanol for 10 min, then washed in H_2_O, and transferred to culture dishes. Both grids and dishes were coated with 1 mg/ml poly-L-lysine (in water or 0.1 M borate buffer) for 1 day, washed (in culture medium or water) and placed in culture medium. Grids were always immersed in liquid sideways and were not allowed to dry. The animals used here were bred in the Animal facility of the Max Planck Institute of Biochemistry for research purposes. Pregnant Sprague–Dawley rats were anesthetized by CO_2_ and sacrificed by decapitation, and the brains of E18 embryos were dissected. This procedure was approved by the Animal facility of the Max Planck Institute of Biochemistry. It is an accordance with the German legislation and does not require a formal government approval. Hippocampal neurons dissected from embryos were dissociated after incubation for 15-30 min in 0.25% trypsin, as described previously [33, 34]. After washing with D-MEM and 10% FBS, neurons were plated on the poly-L-lysine coated EM grids and on the 35 mm dishes at the density of 15 000 cells/cm^2^, or at 30 000 cells/cm^2^ for those used for focused ion beam milling. The medium was changed to B27 supplemented medium, or in few cases to a glia-conditioned medium, or the neurons were transferred to a dish containing a glial feeder cell layer. Cultures were kept at 37°C in 5% CO_2_. In some of the cultures used to image non-synaptic boutons PSD-95:GFP fusion protein was expressed by Semliki Forest Virus infection [35]. We did not observe any difference between the transfected and the non-transfected boutons. Neuronal cultures used for immuno-fluorescence and those where synapses were recorded were not transfected.

For immuno-flourescence, cells were fixed in 4% paraformaldehyde for 20 min, washed, permeabilized with PBST (0.1% TritonX-100 in PBS) for 10 min, blocked in 1% BSA for 30 min, and incubated with 1:200 anti-Synapsin I (Abcam ab18814) primary antibody and labeled with 1:1000 Alexa Fluor 568 conjugated anti-rabbit secondary antibody (Thermo Fisher A–11011), or incubated with 1:200 anti-PSD95 (7E3-1B8, Abcam ab13552) primary antibody and labeled with 1:1000 Alexa Fluor 488 conjugated IgG secondary antibody (Thermo Fisher A-11004). Imaging was done on Zeiss Axiovert 200M light microscope.

Electrophysiological recordings were performed using a Multiclamp 700B amplifier (Molecular Devices). Data were digitized using a Digidata 1322A (Molecular Devices). Pipettes (3–5 M) were pulled from borosilicate glass (Science Products). Whole-cell recordings were obtained from dissociated neurons visualized using differential interference contrast infrared video microscopy. Neurons were continuously superfused with carbogen (95% O2, 5% CO_2_) gassed artificial CSF (ACSF) containing the following (in mM): 130.9 NaCl, 2.75 KCl, 1.43 MgSO_4_, 2.5 CaCl_2_, 1.1 NaH2PO_4_, 28.82 NaHCO_3_, and 11.1 D-glucose. Glass electrodes were filled with an internal solution containing the following (in mM): 150 Cs-gluconate, 8 NaCl, 2 MgATP, 10 HEPES, 0.2 EGTA, and 5 QX-314, pH 7.2. Neurons were clamped at -70 mV holding potential.

### Cryo-ET

Cultures were vitrified at 9-21 (in most cases at 10–14) days in vitro (DIV) by rapid freezing in liquid ethane cooled by liquid nitrogen using a manual plunger or Vitrobot (FEI). Immediately before plunging, 3 μl of prewarmed, buffer-exchanged, concentrated BSA tracer 10 nm gold (Aurion) was applied to grids to serve as fiducial markers. Grids were stored in liquid nitrogen until they were mounted on a cryo-holder and inserted into a microscope.

Vitrified neuronal cultures plated at 30 000 cells/cm^2^ were thinned by focused ion beam (FIB) using a dual beam microscope Quanta 3D FEG (FEI) equipped with a Quorum PP3000T cryo-system (Quorum Technologies) and an in-house developed open nitrogen-circuit 360° rotatable cryo-stage. Platinum was sputtered on the whole grid, for 60 s at 10 mA, in the PP3000T transfer system before milling. For the standard wedge-milling routine the specimen was tilted such that the incident angle of the Ga^+^ ion beam with respect to the sample surface was 6°. Under this shallow angle, the milling was performed in three steps of sequentially decreasing ion beam current at 30 kV acceleration voltage. The first, rough cut was done with a 40 μm wide rectangle pattern and 0.5 nA beam current. Further thinning was then done with 0.3 nA and 0.1 nA beam current. The final polishing step was preformed at 0.05 nA. All milling steps were monitored by single-scan ion beam images using 100 ns pixel-dwell time, and by electron-beam images using a beam current of 5.9 pA at an acceleration voltage of 5 kV and 5 μs pixel-dwell time.

Electron images were recorded on FEI Polara and Philips CM300 electron microscopes, each equipped by a field emission gun (operated at 300 kV), a computerized stage and a post-column energy filter operated in the zero-loss mode (Gatan). Images were recorded on 2kx2k MegaScan charge-coupled device (CCD) camera (Gatan). One tomogram was recorded on Titan Krios microscope (FEI) with K2 Summit direct electron detector device (Gatan). Tomographic series were recorded using Xplore3D (FEI), SerialEM and custom-made low dose acquisition schemes [36, 37]. Angular increment was 1.5-2° and the typical angular range was -60° to + 60°. Three dual-axis tomograms were recorded at 2° increment and the angular range for the second axis was -60° to + 40°. The underfocus was 7-9 μm, the total electron dose was kept <100 e^-^/Å^2^ and the pixel size at the specimen level was 0.81-0.82 μm except in few cases 0.71 nm and for the tomogram acquired on K2 camera 0.42 nm. Tomograms were aligned based on fiducial markers, binned 2 times (bin factor 4; final pixel size 3.2 nm for most tomograms, 2.9 and 1.7 nm for the other tomograms, as mentioned above) and reconstructed using weighted back-projection as implemented in Imod, TOM and EM software packages [38–40]. Tomogram thickness ranged from 200 to 500 nm.

### Image selection and processing

We recorded 77 tomograms from more than 20 culture preparations without FIB milling that were of sufficient technical and biological quality. Specifically, tomograms were deemed technically acceptable if they did not contain any signs of ice crystal formation such as ice reflections or faceted membranes, and they had reasonable signal-to-noise ratio and proper tomographic alignment Boutons were judged biologically relevant by the presence of microtubules and some other organelles such as smooth endoplasmic reticulum, synaptic or larger spherical vesicles or endosomes and absence of aggregated, apoptotic-like material. Among these, 61 boutons that did not form synapses were classified as large axonal boutons (having a width of 400 nm or more and at least two times larger than the neighboring axonal shaft). Furthermore, 38 contained more than ten synaptic vesicles and were thus classified as nonsynaptic axonal boutons. In addition, four tomograms of synapses were obtained from three culture preparations after thinning by FIB. Brightness and contrast was adjusted on some of the tomographic slices used for figures. These adjustments were linear and were applied uniformly on the whole images.

3D segmentation used for visualization of entire boutons and for further processing were done in Amira (FEI) and using the actin segmentation tool [41]. Tomograms selected for further analysis (12 selected as technically the best among nonsynaptic boutons, five of them contained DCVs, as well as three synapses) were denoised using Anisotropic nonlinear diffusion [42] as implemented by [43], using the same parameter values as before [44]. Automated detection and analysis of synaptic vesicle tethers and connectors (2-bound segments) was done using the Hierarchical connectivity segmentation implemented in the Pyto software package, as explained before [44]. Synaptic vesicle and DCV protrusions (1-bound segments) were segmented at a single threshold level (mean of the vesicle membrane density) so that their number and length could be properly compared, again using the Pyto package. To avoid false positives, segments smaller than 3 voxels and larger than 320 nm^3^ were discarded, as it was done in our previous publications. Synaptic vesicle occupancy was measured as the fraction of cytoplasmic volume occupied by synaptic vesicles at different distances to the plasma membrane (using 1 pixel thick layers parallel to the plasma membrane). Only the regions of plasma membrane in the proximity of synaptic vesicles were included for the calculation of the number of vesicles per unit plasma membrane surface area. Protrusion length was calculated using the B-max mode, while tether and connector length was measured membrane edge-to-edge (length mode B2C) [44]. This differs slightly from the C2C mode used in the previous studies, in comparison the values obtained here are up to 1 pixel larger.

#### Experimental design and statistical analysis

The distribution of the microtubule number in axons was analyzed using the zero-truncated Poisson distribution (see below). Statistical analysis between experimental groups was performed using Chi-square test when the data was expressed as frequencies and by t-test otherwise.

#### Zero-truncated Poisson distribution

Theoretical, zero-truncated Poisson distribution was calculated in the standard manner:

$$\begin{array}{c} P\left(k\right)=\frac{\lambda^{k}}{(e^{\lambda }-1)k!} \end{array}$$

where parameter λ was calculated from the experimentally determined mean number of microtubules per axon (*m*) by solving:

$$\begin{array}{c} m=\frac{\lambda }{1-e^{-\lambda }} \end{array}$$

using Brent’s algorithm as implemented in SciPy. Because the experimental number of boutons that contained any number of microtubules higher than 5 was low, the data was binned for 6-8 and more than 8 microtubules. This data was statistically compared with the zero-truncated Posisson distribution using the G-test (log-likelihood ratio test):

$$\begin{array}{c} G=2\;\sum^{n}f_{i}\;ln\left(\frac{f_{i}}{{\hat{f}}_{i}}\right) \end{array}$$

where *f*_*i*_ and ${\hat{f}}_{i}$ are the observed and the expected (in this case zero-truncated Poisson) frequencies for *i*-th bin. The G-value was then compared with χ^2^ distribution using *n* − 1 degrees of freedom to get the probability that the frequencies (number of microtubules per bouton) are not randomly (Poisson) distributed.

#### Microtubule curvature

The results obtained by the microtubule curvature determination method used in [45] depend on the distance between the points used to determine the curvature [46], while the standard way to express curvature (used here) is the inverse of the radius. In order to allow direct comparison, we converted curvatures obtained in our measurements using the reported distance between points of 0.5 μm [45]. Specifically, the curvature of 2.4 μm^-1^ from our data corresponds to 2.5 rad/μm and 1.8 μm^-1^ from our data corresponds to 1.9 rad/μm in that paper.

#### Radial density traces of medium-sized vesicles

Vesicles (lumen and membrane together) were segmented manually and smoothed (magnified 4 times and interpolated using cubic splines). In order to make density traces, concentric shell layers of 1 pixel thickness were made from the vesicle surface both on the vesicle and the cytoplasmic sides and the mean density was calculated for each shell.

#### Computational aspects

All computations were implemented in the Python programming language using Pyto, Numpy, Scipy and Matplotlib scientific computing packages and Ipython / Juputer environment [44, 47–49] and were executed on Linux workstations.

## Results

### Cryo-ET of dissociated neuronal cultures

**Fig 1:**
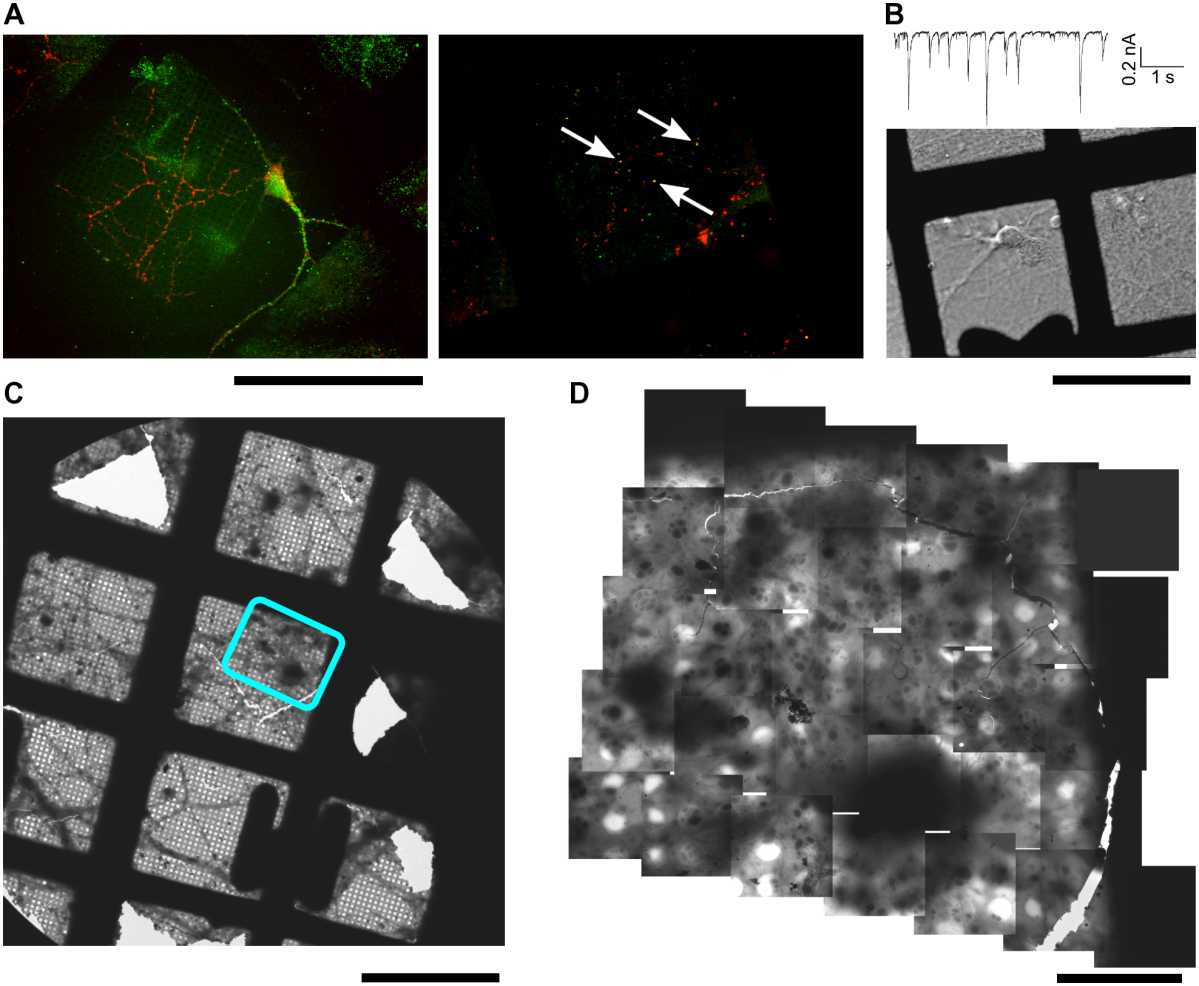
Neurons grown on EM grids. (A) Immuno-fluorescence staining of neurons at DIV 9 (left) and 15 (right) against PSD95 (green) and synapsin (red). Arrows point to the colocalized spots. (B) A representative whole cell trace and the neuron used for the recording. (C) Low magnification EM image of a neuronal culture grown on an EM grid. (D) Collage of medium magnification EM images covering a region indicated on (C). Scale bars A-C 100 μm, D 10 μm.

Although neuronal cultures have already been observed by cryo-ET, the evaluation of this method is still lacking [31, 32, 50–53]. We detected extensive immuno-fluorescence staining for the presynaptic marker Synapsin I on hippocampal neurones grown on standard EM grids. At DIV 15, punctate staining on neurites was obtained for the postsynaptic marker PSD-95, which to an extent colocalized with the Synapsin I staining (Fig 1A, arrows). Furthermore, we recorded synaptic currents elicited by spontaneous occurring action potentials (Fig 1B). Amplitude and kinetics of these events were normal. Further holding currents at -70 mV were smaller than 50 pA indicating a healthy resting potential. Together, these results show that neurons grown on grids develop normally and form synaptic connections.

**Fig 2:**
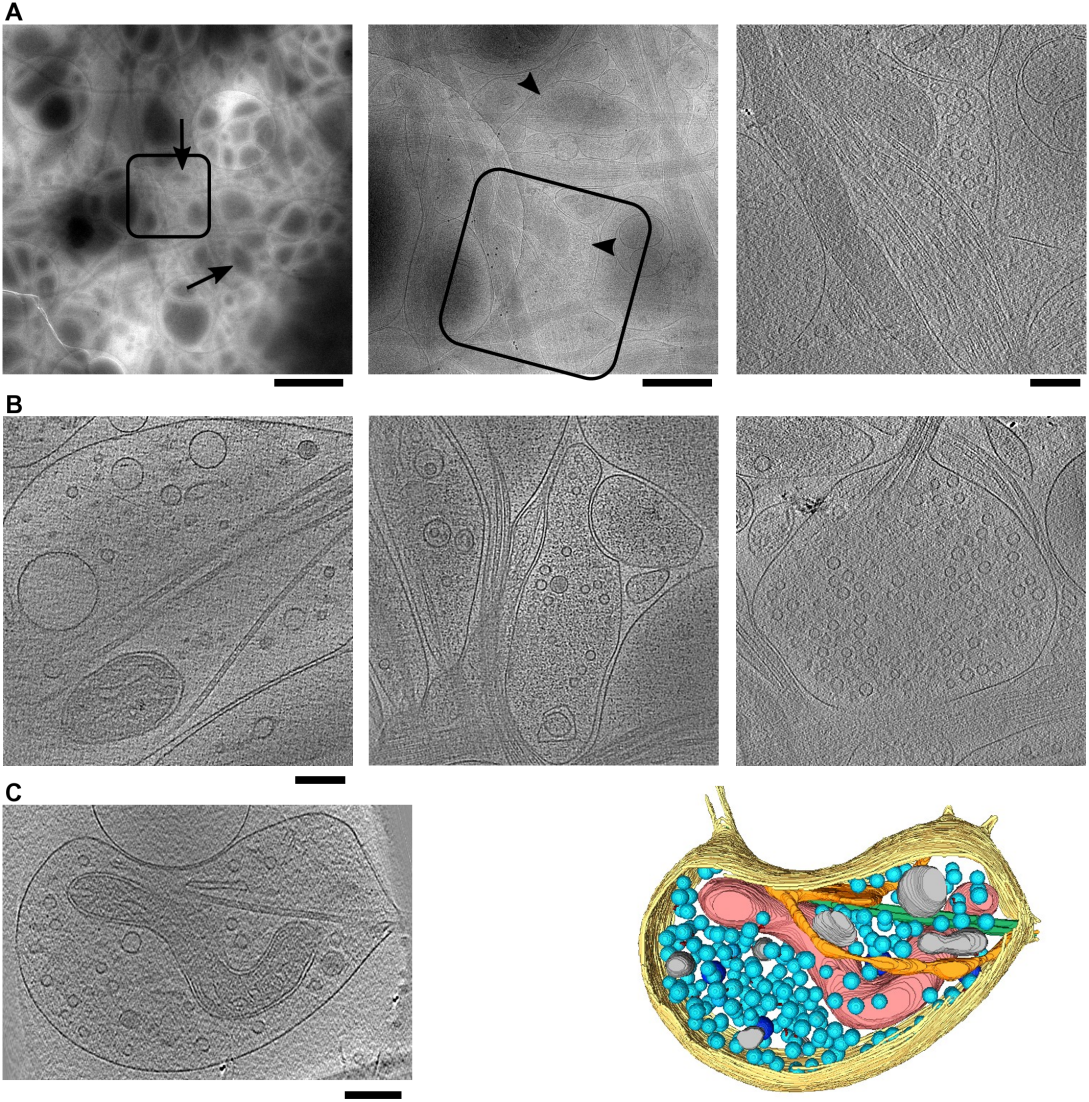
Large axonal bouton types. (A) Images at increasing magnifications leading to an axonal bouton where a tomogram was recorded. Squares show the location of the image on their right. On the left and in the middle are projection images, while the right one is a 9.6 nm thick tomographic slice. Arrows indicate some of neuronal swellings, arrowheads point to synaptic vesicle clusters. Scale bars 2 mm (left), 500 nm (center) and 200 nm (right). (B) Spindle P/T (left), sack S/C (middle), sack S (left) bouton types. (C) A tomographic slice and a 3D segmentation of a bouton containing plasma membrane (yellow), mitochondrion (red), microtubules (green), SER (orange), synaptic vesicles (light blue), dense core vesicles (dark blue) and other membranous compartments (grey). Tomographic slices on (B) are 3.2 nm and on (C) 2.8 nm thick and the scale bares are 200 nm.

EM imaging of vitrified neurons showed that they spread their processes over the grids (Fig 1C and 1D). Neuronal processes often formed bundles, as well as fine sheet-like networks. Upon closer examination, individual neurites could be detected within the regions of thin vitreous material (up to 500 nm in thickness). For tomography, we targeted swellings on neurites, readily detected at medium magnifications (Fig 2A). At increased magnifications, intracellular structures like synaptic vesicles and microtubules were observed. Because cellular structures often overlap in 3D and the electron dose available for such images is limited, many cellular components could only be revealed in reconstructed tomograms (Fig 2B and 2C).

General features such as smooth, continuous membranes, non-extracted cytoplasm and the absence of ice-crystallization demonstrate the faithful preservation provided by cryo-ET (Fig 2). These results show that dissociated neurons can grow properly on EM grids at least up to DIV 14, and confirm that cryo-ET allows imaging of intact, unperturbed neuronal cultures.

### Morphology of axonal boutons

Large axonal boutons (see the Methods) were classified by two orthogonal criteria. First, the bouton shape was described as spindle (roughly symmetric in respect to the main, microtubules defined, axis of the axon) (Fig 2B left and Fig 2C) or sack-like (asymmetric) (Fig 2B center and right). Second, the boutons were categorized by their vesicle content into those containing spherical vesicles (S-type), predominantly flat or elongated (F-type), pleomorphic (P-type), spherical and dense core (S/C-type), elongated and dense core (F/C-type) and pleomorphic vesicles and tubules (P/T types) [54]; SynapseWeb http://synapseweb.clm.utexas.edu/).

Large axonal boutons present in our tomograms were classified based on the vesicle content, confirming that these boutons fit well within the previously established criteria (Table 1). For example, spindle P/T, sack S/C, sack S and spindle S/C boutons are shown on panels B left, middle, right and c of the Fig 2, respectively. Interestingly, we did not observe boutons where flattened or elongated synaptic vesicles predominate. This reduction in vesicle heterogeneity might be explained by different vesicle content, considering recent findings that aldehyde fixation causes flattening of synaptic vesicles that have low osmolarity [55, 56]. While the synaptic vesicles were found in the majority of both spindle and sack-like terminals (S and S/C types taken together), the sack-like were significantly more likely to contain synaptic vesicles than the spindles (t-test, p = 0.018), which might indicate that developing a sack-like shape is a step in the maturation towards the presynaptic boutons.

**Table 1:** Classification of large nonsynaptic axonal boutons according to their shape and content.

	S	S/C	F	F/C	P	P/T	Other	Total
Spindle	8	7	0	0	9	3	2	29
Sack	9	7	0	0	2	0	3	21
Other	2	2	0	0	5	2	0	11
Total	19	16	0	0	16	5	5	61

Spindle and sack refer to the overall shape of boutons, while the columns define the vesicle content: S for synaptic, C dense core, F flattened and P pleomorphic. “Other” denotes boutons that could not be unambiguously categorized.

Other cellular structures, such as microtubules, smooth endoplasmic reticulum (SER), spherical and pleomorphic vesicles were readily identifiable in tomographic slices. Their organization and precise localization can be appreciated in 3D segmentation of complete boutons (Fig 2C) and they are further investigated in the following sections.

### Cytoskeleton

**Fig 3:**
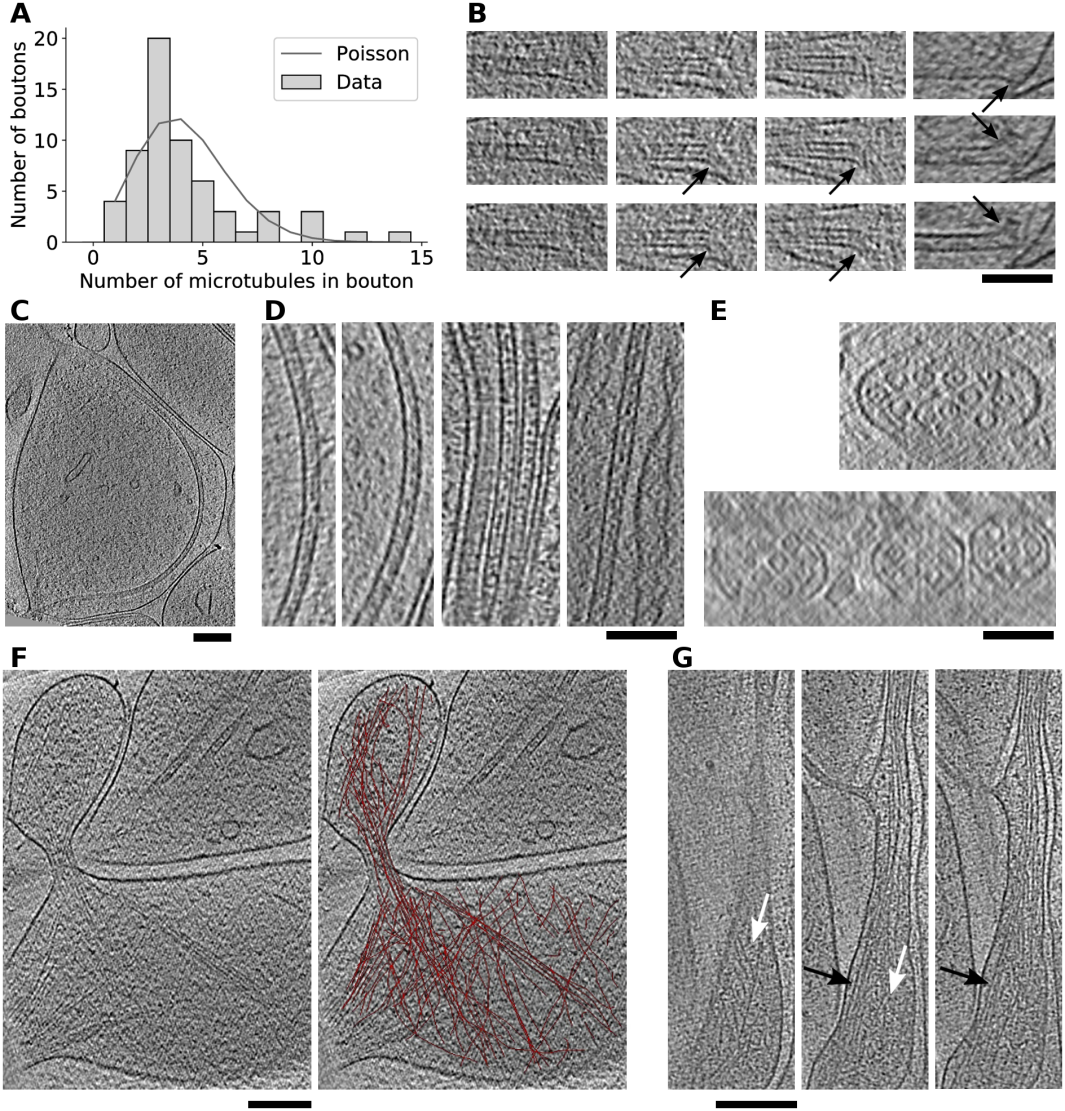
Cytoskeleton. (A) Distribution of the number of microtubules in axonal boutons. (B) Four microtubule ends, each shown on three tomographic slices, some showing frayed ends (arrows). (C) Tomographic slice (9.6 nm thick) showing highly curved microtubule. (D) Lumenal density in microtubules, the first two images on the left are from the microtubule shown on (C). (E) Cross-section of axons, seen as side view tomographic slices, slice thickness is 6.4 (top) and 9.6 nm (bottom). (F) Actin filaments (left) and a 3D segmentation of actin filaments superimposed on the same slice (right). (G) A region of a filopodia at different depths, spanning 29 nm showing cortical actin (white arrows) and bundles (black arrows). Tomographic slices on (B), (D) and (G) are 3.2 nm and on (F) 2.8 nm thick. Scale bars B-E 100 nm and F-G 200 nm.

The mean number of microtubules per large axonal bouton was 4.2, with a sharp peak at 3 (σ = 2.7, N boutons 61, N microtubules 257) (Fig 3A). The distribution of the number of microtubules in the boutons was significantly different from a random distribution (G-test, p = 0.00063, N boutons 61, N microtubules 257, comparison with the zero-truncated Poisson distribution, see Methods). This deviation between the experimental and the random (Poisson) distribution indicates that there was an interaction that clusters microtubules.

While microtubules were separated from each other within axonal boutons (Fig 2), they were tightly bundled in axonal shafts with very little cytoplasmic space between them and the plasma membrane (Fig 3E). The distance between neighboring microtubules was between 10 and 20 nm (measured between wall centers), which is somewhat smaller than 20-30 nm previously reported for cerebellar parallel fiber and spinal cord axons [57]. This tight spacing may be related to the interaction that clusters microtubules.

In reconstituted systems, straight and frayed microtubule ends were associated with growing and depolymerizing microtubules, respectively, but this distinction was less clear in intact cells [58–60]. Microtubule ends detected in our tomograms had both straight and frayed ends (Fig 3B) and were similar to earlier cryo-ET observations in fibroblasts [60]. In addition, some ends were straight on one side and frayed on the other, arguing that individual protofilaments at an end of a microtubule can be in different states.

In our tomograms, several microtubules were highly curved. For example, the curvature of the microtubule shown on Fig 3C was determined to be 1.9 μm^-1^ (corresponding to the radius of the arc formed by the microtubule of 530 nm), while the highest curvature we measured was 2.4 μm^-1^. Compared to the curvatures reported earlier at the leading edge of fibroblasts just before breaking (Fig 2 in [45], 10 of the highest curvatures we measured (all above 1.8 μm^-1^) would fall among the 10 highest curvatures in that paper, when converted to the particular system used for the curvature determination there. While we can not prove that highly curved microtubules exist in vivo, our results show that neuronal microtubules are flexible enough to sustain high curvatures.

Density inside microtubules was detected in cryo-ET slices in immature neurons and astroglia and to a lesser extent in other cell types, and it was implicated in microtubule stability [32, 50, 60–62]. As in the earlier studies, lumenal densities were abundant in our tomograms, they were both disconnected and attached to the microtubule wall and they were prominent at microtubule ends (Fig 3B–3D). Considering that neuronal microtubules can be quite long and that in our images the lumenal density was present in regions of high curvature, it is plausible that the lumenal density provides the required strength or flexibility.

Actin filaments were imaged by cryo-ET in neurons [32, 51] and other cells [63–65]. We observed actin bundles and cortical actin in a protrusion, while a tight bundle was present at its neck (Fig 3F). Criss-crossing filaments at the base likely formed an actin patch, a transient accumulation of actin filaments from which filopodia are known to emerge [66, 67].

We observed a specific form of actin organization in a long (at least 2 μm) filopodium, where tight actin bundles and cortical actin containing nearly parallel branches were found in close proximity, within 30 nm (Fig 3G). The mean spacing between actin filaments in a bundle was 9.6 nm (σ = 1.46 nm, N = 101), similar to that found for reconstituted, fascin cross-linked bundles (8-9 nm, [68] and slightly smaller that the values reported for bundles in cryo-tomograms of Ptk2 cells (12-13 nm, [65] and stained, reconstituted fimbrin cross-linked bundles (11.5-12 nm, [69].

Therefore, the cytoskeletal features observed in our tomograms are in agreement with the literature and the amount of structural detail is at the level of other cellular cryo-ET studies.

### Large membranous compartments

**Fig 4:**
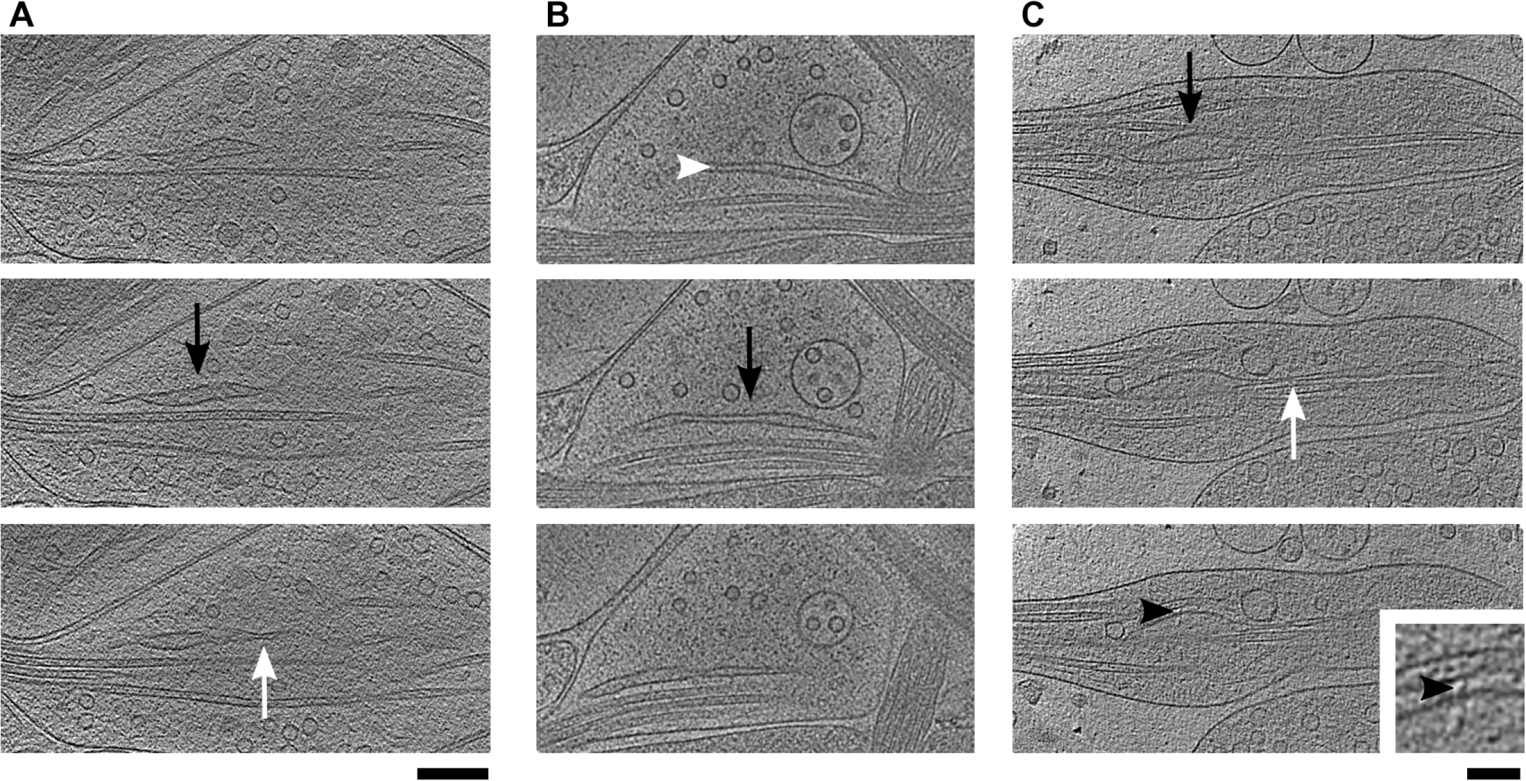
SER from three different boutons. (A) Distance between the first and the last slice is 19 nm. (B) White arrowhead points to a bridge within sack. The distance between slices is 58 nm. (C) Black arrowheads points to a bridge between a SER and a microtubule, a magnified view is shown in the inset. The distance between slices is 26 nm. White arrows point at tubules and black arrows at sacks of SER (all panels). All tomographic slices are 3.2 nm thick. Scale bar for all large slices 200 nm. Inset scale bar 50 nm.

SER was readily observed in our tomograms of axons. As expected, it extended through neuronal processes and was composed of tubules and sacks or sheets (Fig 4, white and black arrows, respectively). A full 3D inspection allowed us to appreciate different forms of sack shapes. The dimensions along the principal axes (length, width and height) of the sack shown on panel (B) were approximately 700, 250 and 20-40 nm, qualifying it as a large, thin sack, while the one on panel (C) was significantly thicker (300, 200 and 80 nm). Because the width of the sack on panel (A) was much closer to its height than to its length (300, 80 and 50 nm), it could be described as a narrow sack or a large, somewhat flattened tubule.

It was proposed that membrane-bound protein bridges act to stabilize the sack shape [70], but such structures were previously detected only in a limited number of micrographs containing sacks from fully hydrated HEK293 cells [71]. Similarly, lumenal bridges that traverse sacks were rare in our data. Few were detected in a thin sack (Fig 4B, white arrowhead), but they were lacking in the thicker ones. Therefore, our data does not support a dominant role of such bridges in maintaining sack shape.

We did not observe direct contact between SER and other lipid membranes. Furthermore, SER and microtubules were rarely linked by protein bridges, despite their proximity (one such bridge is shown on Fig 4C). This observation might explain the finding that the depolymerization of microtubules does not acutely disturb the SER [72].

**Fig 5:**
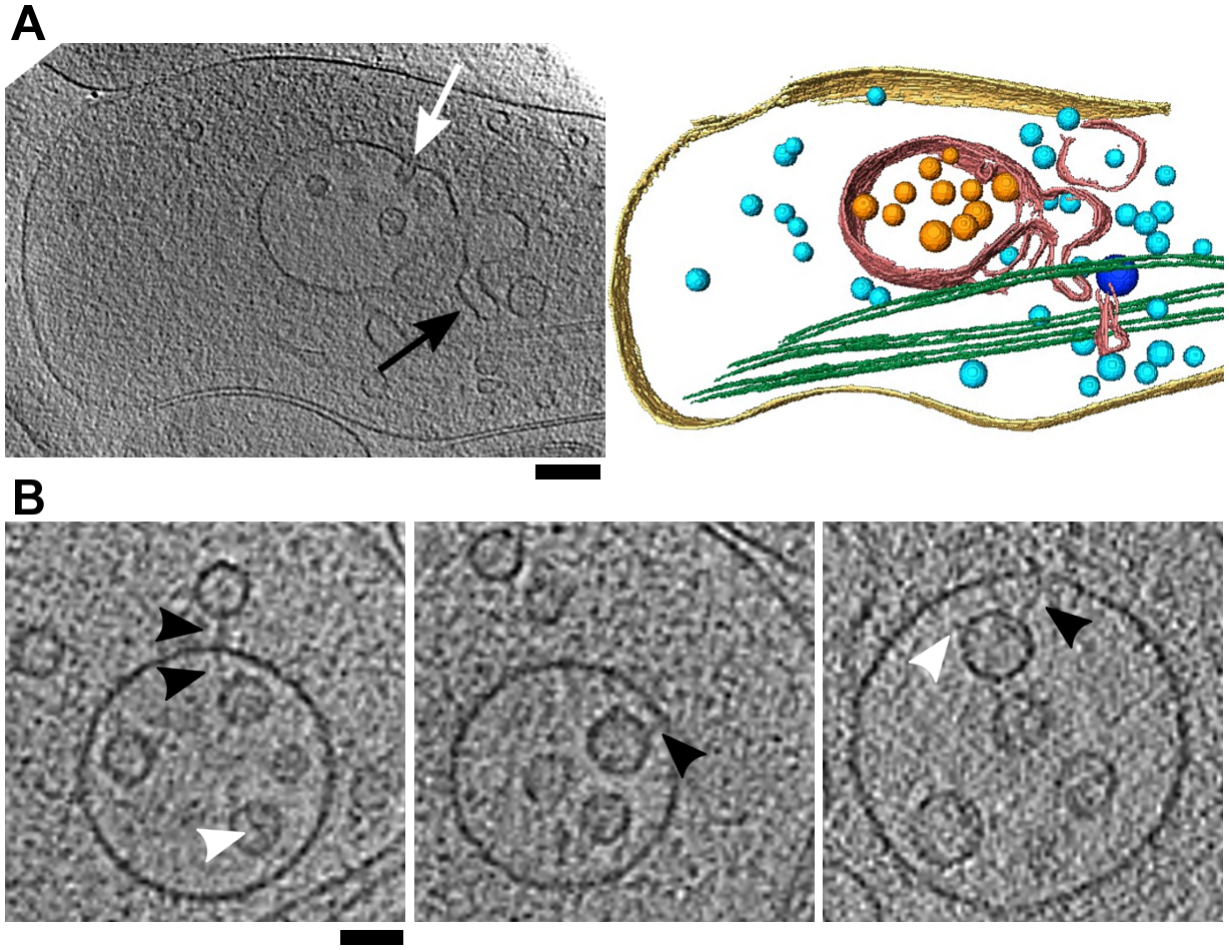
Multivesicular bodies. (A) Tomographic slice and a 3D segmentation of a bouton containing a multivesicular body (red), intralumenal particles (orange), microtubules (green), small vesicles (light blue), medium size vesicles (dark blue) and plasma membrane (yellow). A tubular protrusion is denoted by a black arrow and an internal bud by a white arrow. Scale bar 200 nm. (B) Multivesicular bodies showing intralumenal and external vesicle tethers (black arrowheads) and membrane-bound complexes of intralumenal vesicles (white arrowheads). Scale bar 50 nm. All tomographic slices are 3.2 nm thick.

Multivesicular bodies (MVBs) are single-membrane enclosed compartments containing intralumenal vesicles, and are functionally characterized as late endosomes [73]. We observed more than 20 MVBs, their size ranged from 100 to 400 nm. They showed a smooth, rounded appearance having a stretched membrane (Fig 5), in agreement with earlier results obtained by ET of high-pressure frozen, freeze substituted cells, and in contrast to those from aldehyde-fixed samples [25]. In few cases, the MVBs contained tubular protrusions, external or internal buds (Fig 5A), confirming that they are dynamic structures.

In most cases MVBs contained 5-15 intralumenal vesicles of diameters between 40 and 70 nm (mean±std 52.4±9.6 nm, N = 136), consistent with previous data [74]. We did not observe direct, membrane-to-membrane contact between the intralumenal vesicles and the MVB outer membrane, supporting the view that these vesicles may only transiently contact the MVB outer membrane directly [74]. Instead, several intralumenal vesicles and at least one small cytoplasmic vesicle were tethered to the outer MVB membrane by thin, pleomorphic filaments (Fig 5B, black arrowheads). In addition, intralumenal vesicles contained other membrane-bound structures, protruding to the vesicle exterior as well as to the vesicle lumen side (white arrowheads). Therefore, intralumenal vesicles carry protein complexes that allow them to interact with the outer membrane and may have a role in endosomal function.

### Medium-sized spherical vesicles

**Fig 6:**
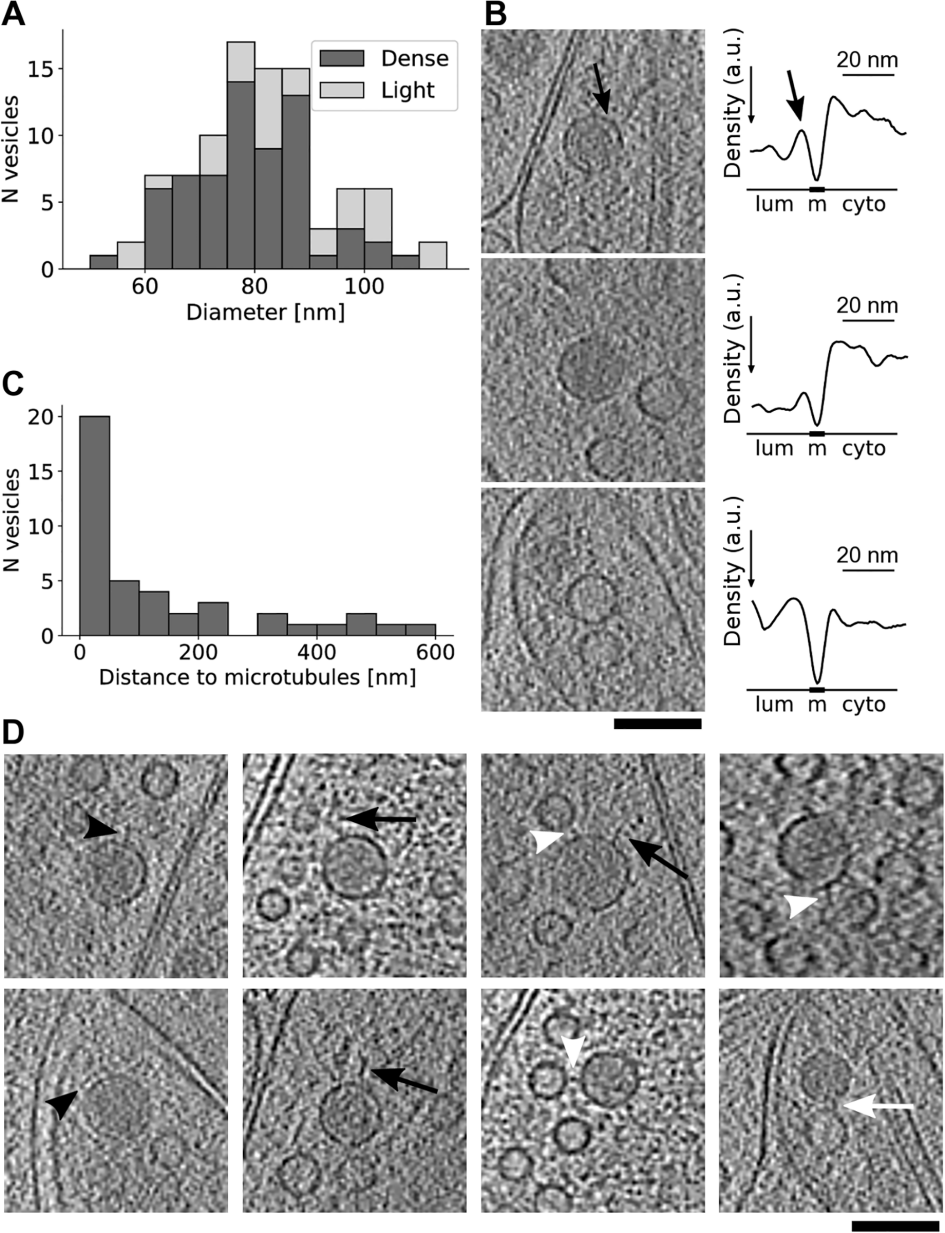
Dense and light core medium size vesicles. (A) Diameter distribution for dense and light core medium size vesicles. (B) Tomographic slices and corresponding radially averaged density traces (lower values indicate higher density) for a dense core with halo (top), a dense core without halo (middle) and a light core vesicle (bottom). Arrows show the light halo and the corresponding peak, while ‘lum’, ‘m’ and ‘cyto’ denote the position of vesicle lumen, membrane and the surrounding cytoplasm, respectively. Scale bars for traces are shown above them. (C) Distance to the nearest microtubule for all dense core, 80 nm vesicles. (D) Tomographic slices showing dense core vesicle protrusions (long black arrows, short black arrowheads), connectors to synaptic vesicles (white arrowheads) and connectors to other medium size vesicles (white arrow). All tomographic slices are 3.2 nm thick. Scale bars for all tomographic slices 100 nm.

More than a half of the medium-sized spherical vesicles (diameter 50-120 nm) that we observed in axons had dark lumen, while the others had light lumen with a density similar to the surrounding cytoplasm (Fig 6B, left). The diameter of the dark lumen vesicles (mean±std 81.0±16.2 nm, N = 66) was significantly smaller than that of the light lumen vesicles (mean±std 94.8±26.1 nm, N = 33; t-test, p = 0.0017) (Fig 6A), which likely indicates they are functionally different.

Further distinction within the dark lumen vesicles was made based on the existence on the light shell (halo) between the vesicle membrane and the core (Fig 6B). Diameters of these two types of dark lumen vesicles did not differ (mean±std with halo 82.7±18.8 nm, N = 31; without halo 79.5±13.7 nm, N = 35). Mean radial density traces of vesicles and the surrounding cytoplasm confirmed the distinction between the three types of medium sized vesicles (Fig 6B, right). Namely, all traces had minima (highest density) at the membrane. The dark lumen vesicle showed lower values (higher density) for lumen than for the surrounding cytoplasm, as opposed to similar values for the light core vesicle. Finally, the light halo surrounding the dense lumen showed as a prominent peak (low density) on the density trace. The different densities described here were not caused by imaging conditions, because tomograms were recorded under similar conditions and the three vesicles shown on Fig 6B were found in the same tomogram.

Dense core vesicles (DCVs) having a mean diameter of 80 nm were previously observed in heavy-metal stained samples and were characterized as vesicles transporting the presynaptic active zone components [1, 4, 75, 76]. Because neuropeptide-containing vesicles also have a dense core but their mean diameter is typically larger than 100 nm [4, 77, 78], we selected dark lumen vesicles having the diameter between 70 and 90 nm. 33% (20 of 61) of large axonal boutons contained DCVs, compared to 17-26% reported earlier [79]. About half of the DCVs (20 of 42) were located within 50 nm to the microtubules (Fig 6C), suggesting that some of the DCVs were transported to boutons. Together, these observations indicate that boutons investigated here may be less mature than those found in tissue.

We found that the DCV membranes were highly decorated by densities, likely composed of proteins (Fig 6D). Among them, free-standing protrusions showed different shapes, such as an array of short protrusions (black arrowhead on the lower left image), individual short (black arrowhead on the top left image), or long protrusions (black arrows). Furthermore, we observed short filaments linking DCVs to other medium size vesicles (white arrow) and synaptic vesicles (white arrowheads), similar to those interlinking synaptic vesicles reported before [22] (quantification is presented in one of the following sections). These DCV attached densities are likely to be involved in the synaptic vesicle organization and axonal transport and may comprise some of the cargo transported by DCVs.

### Transport packets

Axonal boutons containing DCVs and light lumen vesicles, indicative of axonal transport, showed different size and organization.

**Fig 7:**
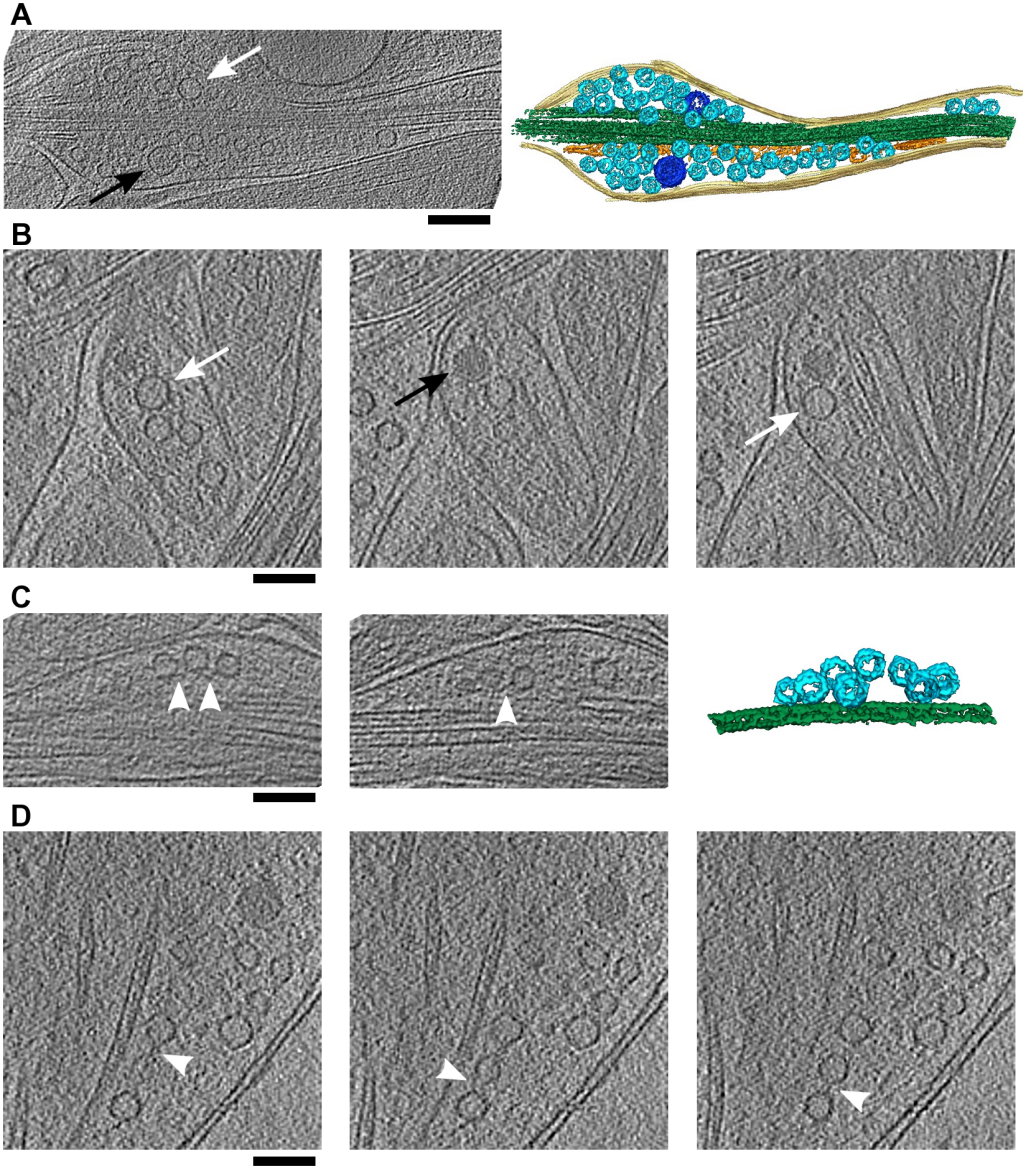
Transport packets. (A) A tomographic slice and a 3D segmentation of a medium-sized bouton. Plasma membrane is shown in yellow, microtubules in green, SER in orange, synaptic vesicles in light blue and medium size vesicles in dark blue. (B) Tomographic slices showing a small transport bouton. Distance between the left and the right slice is 41.6 nm. (C) Short, chain-like clusters of synaptic vesicle in a small bouton. Distance between slices is 38.4 nm. (D), Synaptic vesicle chain in a large bouton. Distance between slices is 9.6 nm. On all panels black arrows indicate DCVs, white arrows light lumen medium size vesicles and white arrowheads connectors interlinking synaptic vesicles. All tomographic slices are 3.2 nm thick. Scale bars (A) 200 nm, (B)-(D) 100 nm.

The first example is a medium-sized bouton, containing more than 30 synaptic vesicles (Fig 7A). The tight packing of the synaptic vesicles and their clustering close to microtubules set this bouton apart from large axonal boutons (Fig 2). Its size, the spindle shape and the presence of a DCV and a light lumen medium sized vesicle, point to a less mature state and support the view that this bouton forms a transport package.

We observed several small boutons that contained a mix of DCVs, light lumen medium sized vesicles and synaptic vesicles (Fig 7B). The vesicles were clustered together, close to microtubules. The composition of these boutons agrees with those proposed to transport the active zone and the synaptic vesicle proteins, except that we did not observe cytoplasmic aggregates [7], probably because of a higher resolving power of cryo-ET.

Interestingly, we also observed small clusters of closely spaced synaptic vesicles, organized in linear, chain-like structures (Fig 7C and 7D). An occasional vesicle was often found slightly off the axis formed by other vesicles (two vesicles on the middle image of panel (C) and the middle vesicle on panel D). Vesicles were typically arranged along microtubules and were located both in small boutons that did not contain other vesicles and also within larger boutons. In both cases these vesicles were interlinked by short filaments, suggesting they were transported together.

Therefore, our data support different modes of axonal transport. Considering that these boutons were wider than the surrounding axonal shafts, their transport necessitates remodeling of the plasma membrane along their path.

### Synaptic vesicles, connectivity and tethering

To investigate the distribution and organization of synaptic vesicles at nonsynaptic axonal boutons in detail, we focused on the technically best tomograms (see the Methods). These included both spindle and sack-like boutons and contained between 30 and more than 200 vesicles.

**Fig 8:**
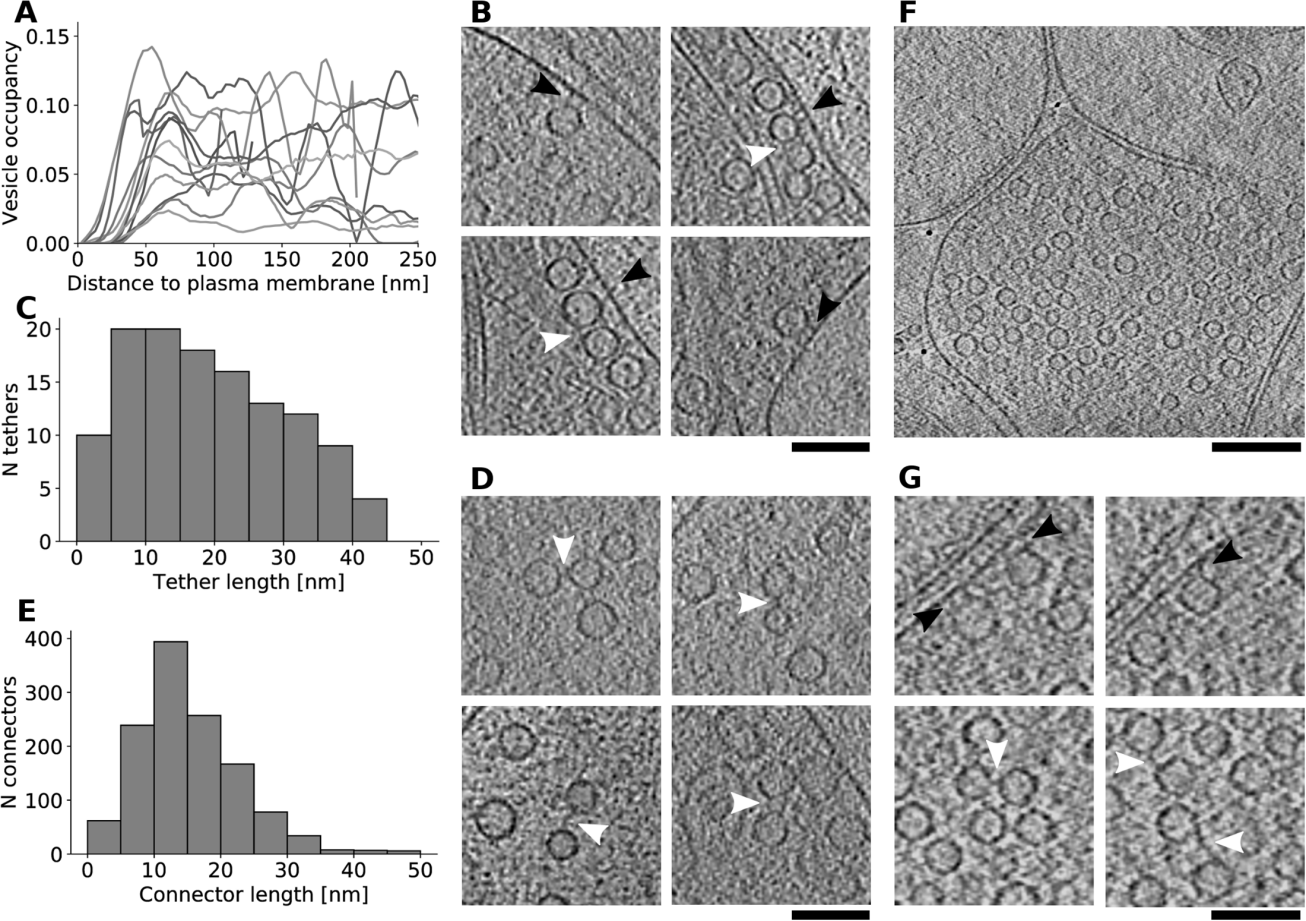
Synaptic vesicle organization, tethers and connectors. (A) Synaptic vesicle distribution in respect to the plasma membrane for individual nonsynaptic boutons. (B) Synaptic vesicle tethers (black arrowheads) and connectors involving tethered vesicles (white arrowheads) in nonsynaptic boutons. (C) Histogram of tether lengths. (D) Synaptic vesicle connectors (white arrowheads) in nonsynaptic boutons. (E) Histogram of synaptic vesicle connector lengths. (F) Tomographic slice of a synapse. (G) Tethers (black arrowheads) and connectors (white arrowheads) in a synapse. Tomographic slices are 2.8-3.2 nm thick. Scale bars (B), (D), (G) 100 nm, (F) 200 nm.

Our visual impression that synaptic vesicles were generally removed from the plasma membrane were substantiated by the quantification of the vesicle distribution in respect the membrane (Fig 8A). Namely, the nonsynaptic boutons lacked the increased vesicle concentration in the proximal region (up to 45 nm to the plasma membrane), which was prominent in undisturbed, mature synaptosomes [80], Fig 2B). Furthermore, the number of proximal synaptic vesicles per unit plasma membrane surface area was 19.4±33.1 μm^-2^ (mean±std, N = 12). This is much lower than what was measured for wild-type synaptosomes (100 μm^-2^, Fig 7D in [81] and 103 μm^-2^ in [80] and even for genetically modified synapses known to have reduced release probability (53 μm^-2^ in [80].

As in our previous studies in synaptosomes, tethers linking synaptic vesicles to the plasma membrane, and short filaments interlinking synaptic vesicles (connectors) were visually identified as the most prominent structures organizing synaptic vesicles (Fig 8B and 8D, arrowheads). Furthermore, even in densely packed boutons, no direct membrane contact between synaptic vesicles and the undisturbed plasma membrane was observed (Fig 8B), consistent with our previous reports.

Connectors and tethers were automatically detected using our Hierarchical thresholding procedure. The fidelity of this procedure was established by the agreement of the automatically detected structures with the ground truth and the observation made on perturbed synapses that the distance between membranes does not determine the detection [22, 44].

Synaptic vesicle connectors were present on more than half of the synaptic vesicles (58%), in some cases linking tethered vesicles (Fig 8D and 8B). Their length showed a strong peak between 10 and 15 nm (mean±std 15.5±7.7 nm, N = 1254) (Fig 8E), but overall their shape was heterogeneous. All these measures were consistent with our previous publications.

In order to investigate the nature of vesicle membrane-bound complexes, we compared those emanating from DCVs and synaptic vesicles in the technically best boutons that contained DCVs. The number of free-standing protrusions per vesicle was higher for DCVs (mean±sem 27.4±4.4, N = 9 for DCVs and 10.8±0.2, N = 590 for synaptic vesicles; t-test p = 0.0001), but the number of protrusions per unit membrane area (mean±sem 1.41±0.21 per 1000 nm^2^ for DCVs and 1.59±0.03 for synaptic vesicles) and their length (mean±sem 9.10±0.48, N = 247 for DCVs and 9.65±0.10, N = 6347 for synaptic vesicles) were similar. Because the analysis of the free-standing protrusions required segmentation at a single threshold, it is expected that some protrusions were not detected [44]. To circumvent this problem, we analyzed connectors linking synaptic vesicles to DCVs using the standard, hierarchical thresholding approach. Their length was significantly different (mean±sem 23.0±8.5 nm, N = 15 for DCV—synaptic vesicle and 15.0±9.0 nm, N = 207 for synaptic vesicle—synaptic vesicle connectors, t-test p = 0.0010). Therefore, our data shows that both DCVs and synaptic vesicles have a considerable number of vesicle-attached complexes and indicates that in this context DCVs determine the type of connectors.

Tethered proximal synaptic vesicles were detected only in 5 of 12 boutons, significantly different from WT synaptosomes investigated earlier (p = 0.0050, Chi-square test in comparison to 9/9 synapses in [80] and p = 0.0074 for 8/8 synapses in [81]. The majority of tethers were 5-25 nm long (mean±std 19.8±11.7 nm, N = 124; measured membrane edge-to edge) (Fig 8C). Interestingly, some of the vesicles had more than one tether (Fig 8B upper and lower right images) and some tethers seemed to have a continuation on the extracellular side (panel B, bottom row).

Importantly, reducing the sample thickness by focused ion beam (FIB) milling allowed us to access thicker regions and record tomograms of synapses (Fig 8F). This method allows thinning samples while maintaining them in the vitrified state [82, 83], but is plagued by problems that preclude its routine use [84]. Again, we observed numerous synaptic vesicle tethers and connectors (Fig 8G). Upon visual inspection these were the major structural organizers of the presynaptic terminal and their morphology was consistent with those observed in nonsynaptic boutons and synaptosomes. The fraction of tethered proximal synaptic vesicles (mean±sem 0.78±0.09, N = 50), the number of tethers per proximal synaptic vesicle (1.80±0.21, N = 50) and their length (10.93±0.82 nm, N = 92) were similar to the values previously obtained for untreated wild type synaptosomes (0.55-0.78, 1.0-2.7 and 5-12 nm, respectively) [22, 80, 81]. The inverse correlation between the number of tethers per tethered synaptic vesicle and the vesicle distance to the active zone membrane was significant (Pearson’s test, correlation coefficient r = -0.63, p<0.001), arguing against synaptic release defects. Contrary to the non-synaptic boutons, tethers were found in all presynaptic terminals and their mean length was significantly different between the two bouton types(t-test, p<0.001). This data requires careful interpretation because of the small number of analyzed synapses. It nevertheless suggests that tethers detected in synapses in culture are quantitatively similar to those previously analyzed in mature synaptosomes, but are significantly different from those of the non-synaptic boutons.

## Discussion

We employed cryo-ET to obtain high-resolution, 3D images of intact, fully hydrated, vitrified neuronal processes, preserved in a close-to-native state. Our main finding is that different types of spherical vesicles, including synaptic, transport and endosomal vesicles are extensively interconnected or tethered to other lipid membranes, such as the plasma membrane, via short, pleomorphic linkers. These linkers (connectors and tethers) appear to play major roles in the structural organization of synaptic and transport processes. We also provide structural characterization of other cellular components present in neurites, which can serve as a framework into which information from other methods need to fit.

Here, we first characterized the application of cryo-ET on intact neurons. We showed that dissociated hippocampal neurons develop properly on EM grids and form synapses. Synapses were rarely observed without thinning by FIB, likely because in this case the imaging was restricted to thin regions of distal processes. Nevertheless, we observed many nonsynaptic axonal boutons containing synaptic vesicles and confirmed that they fit well with the already existing morphological and vesicular content-based classification [54]; SynapseWeb http://synapseweb.clm.utexas.edu/). The morphology of the SER and late endosomes was consistent with previous EM results [25, 85, 86]. In particular, lipid membranes remained at a distance from each other, as reported earlier [74, 87]. Therefore, we established that cryo-ET is suitable for high-resolution imaging of dissociated neuronal cultures.

Importantly, smooth and continuous membranes, non-extracted cytoplasm and the absence of crystalline ice ensured proper preservation of our samples. The structure of microtubule ends and the lack of long extensions were in good agreement with the findings obtained for fibroblasts [60], even though imaging of our samples was more challenging due to the higher sample thickness. Also, our data showed that lumenal density in microtubules was quite prominent, in agreement with earlier data from intact neurons [32, 50, 62]. Considering that we detected several microtubules that, up to our best knowledge, had a curvature comparable to the most curved microtubules reported so far [45], it is tempting to make an association between microtubule flexibility and lumenal density. Similarly, actin filament bundles and cortical actin in our tomograms were in agreement with those previously observed by cryo-ET [32, 51, 63–65]. Also, we determined the spacing between filaments in a bundle and detected actin organization where bundles and cortical actin are closely apposed. These results show that our tomograms provide a high level of structural detail and conform with the state-of-the-art cellular cryo-ET.

The following arguments support our view that the majority of DCVs we observed can be functionally identified as PTVs. Their morphology was consisitent with PTVs [1, 2], and they appear to be different from neuropeptide-containing vesicles because their mean diameter was 80 nm, which is smaller than 100 nm or more, which is characteristic of neuropeptide-containing vesicles [1, 4, 77, 78, 88], and they were found at non-synaptic boutons at low frequency. Furthermore, they were decorated with a large number of membrane-attached protrusions. Considering that synaptic vesicles are known to have many heterogenous membrane-bound complexes [89], the lack of significant differences between basic morphological properties of DCVs and synaptic vesicles does not give a definite answer about the molecular identity of the DCV protrusions. However, our finding that connectors linking DCVs with synaptic vesicles were different from those interlinking synaptic vesicles point to different composition of DCV connectors. Finally, the common location of DCVs in the vicinity of microtubules further supports their role in axonal transport.

We observed axonal packets comprising dense and light core medium size vesicles, putatively identified as PTVs and STVs, as well as synaptic vesicles. The mixed composition of these packets supports the view that PTVs and STVs may be arrested at the same spots in axons, or transported together [7–9, 90]. Additionally, we detected short chain-like clusters of synaptic vesicles, often aligned parallel to microtubules which we hypothesize are also involved in transport, thus further supporting multiple modes of axonal transport [9].

Because our samples were not stained by heavy metals, we conclude that the lumen of DCVs indeed contains material of a higher mass density. Furthermore, the variable spatial extent of the dense material, filling the entire lumen or at a distance to the vesicle membrane, argues that the organization or composition of lumenal material differs between DCVs.

Synaptic vesicles in synaptic and nonsynaptic boutons were extensively interlinked by pleomorphic short filaments (connectors) and they were tethered to the plasma membrane. These synaptic vesicle connectors and tethers were the most prominent structural features organizing synaptic vesicles. Their localization and morphology of connectors was consistent with the previous data from synaptosomes that implicated these structure in synaptic vesicle mobilization and release [22, 80].

Quantification based on the automated tether detection in presynaptic terminals by the Hierarchical thresholding [44], agreed with that earlier reported for mature synaptosomes [22, 80, 81]. However, non-synaptic boutons structurally differed from presynaptic terminals in the following ways: (i) There were fewer proximal SVs in the non-synaptic boutons. (ii) Tethered proximal synaptic vesicles were found only in some non-synaptic boutons, as opposed to all synapses. (iii) Tether length was significantly longer in non-synaptic boutons. Together, this argues that nonsynaptic boutons possess some of the required components, but are not fully release-competent. This agrees with findings that nonsynaptic boutons are capable of neurotransmitter release and play a role in synaptogenesis, and that the release requires some SNARE proteins but is not sensitive to tetanus toxin, which abolishes the release at mature synapses [12–16, 91]. Therefore, it is likely that synaptic vesicle connectors and tethers of nonsynaptic boutons are involved in synaptic function, specifically in synaptic vesicle organization and release.

The above findings are consistent with our previous hypothesis that in mature terminals, initially tethered SVs acquire multiple short tethers, likely in a SNARE-dependent manner, during a progression towards the release [22, 80]. However, this points to the possibility that the release involving different SNARE protein isoforms [91] may have a different structural signature.

Importantly, we observed similar connectors and tethers between late endosomes and their intralumenal vesicles, and between dense core vesicles and other medium size or synaptic vesicles both in transport packets and nonsynaptic boutons. Short linkers between other cellular structures, such as microtubules and SER were substantially less prominent. Therefore, our data argues that connectors and tethers are important for the organization of different small and medium sized spherical vesicles and that they are involved in multiple cellular processes.

Taken together, our cryo-ET images of fully hydrated, unperturbed neurons grown in culture show that connectors and tethers link different types of spherical vesicles to other vesicles and lipid membranes, probably serving to organize and precisely position them. They are abundant in neuronal processes and are likely involved in synaptic, endosomal and axonal transport processes. Therefore, structural interactions via short filaments may serve as a ubiquitous form of organization of vesicles in neurons.

## References

[1] Zhai RG, Vardinon-Friedman H, Cases-Langhoff C, Becker B, Gundelfinger ED, Ziv NE, et al Assembling the presynaptic active zone: a characterization of an active one precursor vesicle. Neuron. 2001;29:131–143. doi: 10.1016/S0896-6273(01)00185-4 11182086 10.1016/s0896-6273(01)00185-4

[2] Shapira M, Zhai RG, Dresbach T, Bresler T, Torres VI, Gundelfinger ED, et al Unitary assembly of presynaptic active zones from Piccolo-Bassoon transport vesicles. Neuron. 2003;38(2):237–52. doi: 10.1016/S0896-6273(03)00207-1 12718858 10.1016/s0896-6273(03)00207-1

[3] Dresbach T, Torres V, Wittenmayer N, Altrock WD, Zamorano P, Zuschratter W, et al Assembly of active zone precursor vesicles: obligatory trafficking of presynaptic cytomatrix proteins Bassoon and Piccolo via a trans-Golgi compartment. J Biol Chem. 2006;281(9):6038–47. doi: 10.1074/jbc.M508784200 16373352 10.1074/jbc.M508784200

[4] Sorra KE, Mishra A, Kirov SA, Harris KM. Dense core vesicles resemble active-zone transport vesicles and are diminished following synaptogenesis in mature hippocampal slices. Neuroscience. 2006;141:2097–2106. doi: 10.1016/j.neuroscience.2006.05.033 16797135 10.1016/j.neuroscience.2006.05.033

[5] Ahmari SE, Buchanan J, Smith SJ. Assembly of presynaptic active zones from cytoplasmic transport packets. Nat Neurosci. 2000;3(5):445–51. doi: 10.1038/74814 10769383 10.1038/74814

[6] Bury LAD, Sabo SL. Building a Terminal: Mechanisms of Presynaptic Development in the CNS. Neuroscientist. 2016;22:372–391. doi: 10.1177/1073858415596131 26208860 10.1177/1073858415596131

[7] Tao-Cheng JH. Ultrastructural localization of active zone and synaptic vesicle proteins in a preassembled multi-vesicle transport aggregate. Neuroscience. 2007;150:575–584. doi: 10.1016/j.neuroscience.2007.09.031 17977664 10.1016/j.neuroscience.2007.09.031 PMC2190624

[8] Bury LAD, Sabo SL. Coordinated trafficking of synaptic vesicle and active zone proteins prior to synapse formation. Neural development. 2011;6:24 doi: 10.1186/1749-8104-6-24 21569270 10.1186/1749-8104-6-24 PMC3103415

[9] Petzoldt AG, Lützkendorf J, Sigrist SJ. Mechanisms controlling assembly and plasticity of presynaptic active zone scaffolds. Curr Opin Neurobiol. 2016;39:69–76. doi: 10.1016/j.conb.2016.04.009 27131423 10.1016/j.conb.2016.04.009

[10] Scott DA, Das U, Tang Y, Roy S. Mechanistic logic underlying the axonal transport of cytosolic proteins. Neuron. 2011;70:441–454. doi: 10.1016/j.neuron.2011.03.022 21555071 10.1016/j.neuron.2011.03.022 PMC3096075

[11] Maeder CI, Shen K, Hoogenraad CC. Axon and dendritic trafficking. Curr Opin Neurobiol. 2014;27:165–170. doi: 10.1016/j.conb.2014.03.015 24762653 10.1016/j.conb.2014.03.015

[12] Matteoli M, Takei K, Perin MS, Südhof TC, De Camilli P. Exo-endocytotic recycling of synaptic vesicles in developing processes of cultured hippocampal neurons. J Cell Biol. 1992;117:849–861. doi: 10.1083/jcb.117.4.849 1577861 10.1083/jcb.117.4.849 PMC2289460

[13] Kraszewski K, Mundigl O, Daniell L, Verderio C, Matteoli M, De Camilli P. Synaptic vesicle dynamics in living cultured hippocampal neurons visualized with CY3-conjugated antibodies directed against the lumenal domain of synaptotagmin. J Neurosci. 1995;15:4328–4342. doi: 10.1523/JNEUROSCI.15-06-04328.1995 7540672 10.1523/JNEUROSCI.15-06-04328.1995 PMC6577731

[14] Krueger SR, Kolar A, Fitzsimonds RM. The presynaptic release apparatus is functional in the absence of dendritic contact and highly mobile within isolated axons. Neuron. 2003;40(5):945–57. doi: 10.1016/S0896-6273(03)00729-3 14659093 10.1016/s0896-6273(03)00729-3

[15] Ratnayaka A, Marra V, Branco T, Staras K. Extrasynaptic vesicle recycling in mature hippocampal neurons. Nat Commun. 2011;2:531 doi: 10.1038/ncomms1534 22068598 10.1038/ncomms1534 PMC3492933

[16] Andreae LC, Fredj NB, Burrone J. Independent vesicle pools underlie different modes of release during neuronal development. J Neurosci. 2012;32:1867–1874. doi: 10.1523/JNEUROSCI.5181-11.2012 22302825 10.1523/JNEUROSCI.5181-11.2012 PMC6703344

[17] Landis D, Hall A, Weinstein L, Reese T. The organization of cytoplasm at the presynaptic active zone of a central nervous-system synapse. Neuron. 1988;1(3):201–209. doi: 10.1016/0896-6273(88)90140-7 3152289 10.1016/0896-6273(88)90140-7

[18] Hirokawa N, Sobue K, Kanda K, Harada A, Yorifuji H. The cytoskeletal architecture of the presynaptic terminal and molecular structure of synapsin 1. J Cell Biol. 1989;108(1):111–126. doi: 10.1083/jcb.108.1.111 2536030 10.1083/jcb.108.1.111 PMC2115350

[19] Takei Y, Harada A, Takeda S, Kobayashi K, Terada S, Noda T, et al Synapsin I deficiency results in the structural change in the presynaptic terminals in the murine nervous system. The Journal of Cell Biology. 1995;131(6):1789–1800. doi: 10.1083/jcb.131.6.1789 8557745 10.1083/jcb.131.6.1789 PMC2120677

[20] Gustafsson J, Birinyi A, Crum J, Ellisman M, Brodin L, Shupliakov O. Ultrastructural organization of lamprey reticulospinal synapses in three dimensions. J Comp Neurol. 2002;450(2):167–182. doi: 10.1002/cne.10310 12124761 10.1002/cne.10310

[21] Siksou L, Varoqueaux F, Pascual O, Triller A, Brose N, Marty S. A common molecular basis for membrane docking and functional priming of synaptic vesicles. Eur J Neurosci. 2009;30(1):49–56. doi: 10.1111/j.1460-9568.2009.06811.x 19558619 10.1111/j.1460-9568.2009.06811.x

[22] Fernández-Busnadiego R, Zuber B, Maurer UE, Cyrklaff M, Baumeister W, Lucic V. Quantitative analysis of the native presynaptic cytomatrix by cryoelectron tomography. J Cell Biol. 2010;188(1):145–56. doi: 10.1083/jcb.200908082 20065095 10.1083/jcb.200908082 PMC2812849

[23] Cole AA, Chen X, Reese TS. A network of three types of filaments organizes synaptic vesicles for storage, mobilization, and docking. Journal of Neuroscience. 2016;36(11):3222–3230. doi: 10.1523/JNEUROSCI.2939-15.2016 26985032 10.1523/JNEUROSCI.2939-15.2016 PMC4792936

[24] Dubochet J, Sartori Blanc N. The cell in absence of aggregation artifacts. Micron. 2001;32(1):91–9. doi: 10.1016/S0968-4328(00)00026-3 10900384 10.1016/s0968-4328(00)00026-3

[25] Murk JLAN, Posthuma G, Koster AJ, Geuze HJ, Verkleij AJ, Kleijmeer MJ, et al Influence of aldehyde fixation on the morphology of endosomes and lysosomes: quantitative analysis and electron tomography. Journal of microscopy. 2003;212:81–90. doi: 10.1046/j.1365-2818.2003.01238.x 14516365 10.1046/j.1365-2818.2003.01238.x

[26] Al-Amoudi A, Norlen LP, Dubochet J. Cryo-electron microscopy of vitreous sections of native biological cells and tissues. J Struct Biol. 2004;148(1):131–5. doi: 10.1016/j.jsb.2004.03.010 15363793 10.1016/j.jsb.2004.03.010

[27] Bleck CK, Merz A, Gutierrez MG, Walther P, Dubochet J, Zuber B, et al Comparison of different methods for thin section EM analysis of Mycobacterium smegmatis. J Microsc. 2010;237(1):23–38. doi: 10.1111/j.1365-2818.2009.03299.x 20055916 10.1111/j.1365-2818.2009.03299.x

[28] Li Y, Almassalha LM, Chandler JE, Zhou X, Stypula-Cyrus YE, Hujsak KA, et al The effects of chemical fixation on the cellular nanostructure. Exp Cell Res. 2017;358(2):253–259. doi: 10.1016/j.yexcr.2017.06.022 28673821 10.1016/j.yexcr.2017.06.022 PMC5726765

[29] Dubochet J, Adrian M, Chang JJ, Homo JC, Lepault J, McDowall AW, et al Cryo-electron microscopy of vitrified specimens. Q Rev Biophys. 1988;21(2):129–228. doi: 10.1017/S0033583500004297 3043536 10.1017/s0033583500004297

[30] Lucic V, Rigort A, Baumeister W. Cryo-electron tomography: the challenge of doing structural biology in situ. J Cell Biol. 2013;202(3):407–419. doi: 10.1083/jcb.201304193 23918936 10.1083/jcb.201304193 PMC3734081

[31] Lucic V, Kossel AH, Yang T, Bonhoeffer T, Baumeister W, Sartori A. Multiscale imaging of neurons grown in culture: from light microscopy to cryo-electron tomography. J Struct Biol. 2007;160(2):146–56. doi: 10.1016/j.jsb.2007.08.014 17905597 10.1016/j.jsb.2007.08.014

[32] Fukuda Y, Laugks U, Lučić V, Baumeister W, Danev R. Electron cryotomography of vitrified cells with a Volta phase plate. J Struct Biol. 2015;190(2):143–154. doi: 10.1016/j.jsb.2015.03.004 25770733 10.1016/j.jsb.2015.03.004

[33] Goslin K, Banker G. Rat hippocampal neurons in low-density culture In: Banker G, Goslin K, editors. Culturing nerve cells. MIT Press; 1991 p. 251–81.

[34] Kaech S, Banker G. Culturing hippocampal neurons. Nat Protoc. 2006;1(5):2406–2415. doi: 10.1038/nprot.2006.356 17406484 10.1038/nprot.2006.356

[35] Stein V, House DRC, Bredt DS, Nicoll RA. Postsynaptic density-95 mimics and occludes hippocampal long-term potentiation and enhances long-term depression. J Neurosci. 2003;23:5503–5506. doi: 10.1523/JNEUROSCI.23-13-05503.2003 12843250 10.1523/JNEUROSCI.23-13-05503.2003 PMC6741246

[36] Koster AJ, Grimm R, Typke D, Hegerl R, Stoschek A, Walz J, et al Perspectives of molecular and cellular electron tomography. J Struct Biol. 1997;120(3):276–308. doi: 10.1006/jsbi.1997.3933 9441933 10.1006/jsbi.1997.3933

[37] Mastronarde DN. Automated electron microscope tomography using robust prediction of specimen movements. J Struct Biol. 2005;152(1):36–51. doi: 10.1016/j.jsb.2005.07.007 16182563 10.1016/j.jsb.2005.07.007

[38] Kremer JR, Mastronarde DN, McIntosh JR. Computer visualization of three-dimensional image data using IMOD. J Struct Biol. 1996;116(1):71–76. doi: 10.1006/jsbi.1996.0013 8742726 10.1006/jsbi.1996.0013

[39] Nickell S, Forster F, Linaroudis A, Net WD, Beck F, Hegerl R, et al TOM software toolbox: acquisition and analysis for electron tomography. J Struct Biol. 2005;149(3):227–34. doi: 10.1016/j.jsb.2004.10.006 15721576 10.1016/j.jsb.2004.10.006

[40] Hegerl R. The EM Program Package: A Platform for Image Processing in Biological Electron Microscopy. J Struct Biol. 1996;116(1):30–4. doi: 10.1006/jsbi.1996.0006 8812976 10.1006/jsbi.1996.0006

[41] Rigort A, Günther D, Hegerl R, Baum D, Weber B, Prohaska S, et al Automated segmentation of electron tomograms for a quantitative description of actin filament networks. J Struct Biol. 2012;177(1):135–144. doi: 10.1016/j.jsb.2011.08.012 21907807 10.1016/j.jsb.2011.08.012

[42] Frangakis AS, Hegerl R. Noise reduction in electron tomographic reconstructions using nonlinear anisotropic diffusion. J Struct Biol. 2001;135(3):239–50. doi: 10.1006/jsbi.2001.4406 11722164 10.1006/jsbi.2001.4406

[43] Fernandez JJ, Li S. An improved algorithm for anisotropic nonlinear diffusion for denoising cryo-tomograms. J Struct Biol. 2003;144(1-2):152–61. doi: 10.1016/j.jsb.2003.09.010 14643218 10.1016/j.jsb.2003.09.010

[44] Lucic V, Fernández-Busnadiego R, Laugks U, Baumeister W. Hierarchical detection and analysis of macromolecular complexes in cryo-electron tomograms using Pyto software. Journal of structural biology. 2016;196:503–514. doi: 10.1016/j.jsb.2016.10.004 27742578 10.1016/j.jsb.2016.10.004

[45] Odde DJ, Ma L, Briggs AH, DeMarco A, Kirschner MW. Microtubule bending and breaking in living fibroblast cells. J Cell Sci. 1999;112 (Pt 19):3283–3288. 10504333 10.1242/jcs.112.19.3283

[46] Bicek AD, Tüzel E, Kroll DM, Odde DJ. Analysis of microtubule curvature. Methods Cell Biol. 2007;83:237–268. doi: 10.1016/S0091-679X(07)83010-X 17613311 10.1016/S0091-679X(07)83010-X

[47] Jones E, Oliphant T, Peterson P, et al. SciPy: Open source scientific tools for Python; 2001–. Available from: http://www.scipy.org/.

[48] Hunter JD. Matplotlib: A 2D graphics environment. Computing In Science & Engineering. 2007;9(3):90–95. doi: 10.1109/MCSE.2007.55

[49] Perez F, Granger BE. IPython: A System for Interactive Scientific Computing. Comput Sci Eng. 2007;9(3):21–29. doi: 10.1109/MCSE.2007.53

[50] Garvalov BK, Zuber B, Bouchet-Marquis C, Kudryashev M, Gruska M, Beck M, et al Luminal particles within cellular microtubules. J Cell Biol. 2006;174(6):759–65. doi: 10.1083/jcb.200606074 16954350 10.1083/jcb.200606074 PMC2064330

[51] Maurer UE, Sodeik B, Grünewald K. Native 3D intermediates of membrane fusion in herpes simplex virus 1 entry. Proceedings of the National Academy of Sciences of the United States of America. 2008;105:10559–10564. doi: 10.1073/pnas.0801674105 18653756 10.1073/pnas.0801674105 PMC2492464

[52] Shahmoradian SH, Galiano MR, Wu C, Chen S, Rasband MN, Mobley WC, et al Preparation of primary neurons for visualizing neurites in a frozen-hydrated state using cryo-electron tomography. Journal of visualized experiments: JoVE. 2014; p. e50783 doi: 10.3791/50783 24561719 10.3791/50783 PMC4089403

[53] Asano S, Fukuda Y, Beck F, Aufderheide A, Förster F, Danev R, et al Proteasomes. A molecular census of 26S proteasomes in intact neurons. Science (New York, NY). 2015;347:439–442. doi: 10.1126/science.1261197 10.1126/science.1261197 25613890

[54] Bodian D. An electron microscopic characterization of classes of synaptic vesicles by means of controlled aldehyde fixation. J Cell Biol. 1970;44:115–124. doi: 10.1083/jcb.44.1.115 4982204 10.1083/jcb.44.1.115 PMC2107784

[55] Siksou L, Silm K, Biesemann C, Nehring RB, Wojcik SM, Triller A, et al A role for vesicular glutamate transporter 1 in synaptic vesicle clustering and mobility. European Journal of Neuroscience. 2013;37(10):1631–1642. doi: 10.1111/ejn.12199 23581566 10.1111/ejn.12199

[56] Korogod N, Petersen CC, Knott GW. Ultrastructural analysis of adult mouse neocortex comparing aldehyde perfusion with cryo fixation. Elife. 2015;4 doi: 10.7554/eLife.05793 26259873 10.7554/eLife.05793 PMC4530226

[57] Chen J, Kanai Y, Cowan NJ, Hirokawa N. Projection domains of MAP2 and tau determine spacings between microtubules in dendrites and axons. Nature. 1992;360:674–677. doi: 10.1038/360674a0 1465130 10.1038/360674a0

[58] Mandelkow EM, Mandelkow E, Milligan RA. Microtubule dynamics and microtubule caps: a time-resolved cryo-electron microscopy study. J Cell Biol. 1991;114:977–991. doi: 10.1083/jcb.114.5.977 1874792 10.1083/jcb.114.5.977 PMC2289108

[59] Chrétien D, Fuller SD, Karsenti E. Structure of growing microtubule ends: two-dimensional sheets close into tubes at variable rates. J Cell Biol. 1995;129:1311–1328. doi: 10.1083/jcb.129.5.1311 7775577 10.1083/jcb.129.5.1311 PMC2120473

[60] Koning RI, Zovko S, Barcena M, Oostergetel GT, Koerten HK, Galjart N, et al Cryo electron tomography of vitrified fibroblasts: microtubule plus ends in situ. J Struct Biol. 2008;161(3):459–68. doi: 10.1016/j.jsb.2007.08.011 17923421 10.1016/j.jsb.2007.08.011

[61] Cyrklaff M, Kudryashev M, Leis A, Leonard K, Baumeister W, Menard R, et al Cryoelectron tomography reveals periodic material at the inner side of subpellicular microtubules in apicomplexan parasites. J Exp Med. 2007;204(6):1281–7. doi: 10.1084/jem.20062405 17562819 10.1084/jem.20062405 PMC2118598

[62] Bouchet-Marquis C, Zuber B, Glynn AM, Eltsov M, Grabenbauer M, Goldie KN, et al Visualization of cell microtubules in their native state. Biol Cell. 2007;99:45–53. doi: 10.1042/BC20060081 17049046 10.1042/BC20060081

[63] Medalia O, Weber I, Frangakis AS, Nicastro D, Gerisch G, Baumeister W. Macromolecular architecture in eukaryotic cells visualized by cryoelectron tomography. Science. 2002;298(5596):1209–13. doi: 10.1126/science.1076184 12424373 10.1126/science.1076184

[64] Medalia O, Beck M, Ecke M, Weber I, Neujahr R, Baumeister W, et al Organization of actin networks in intact filopodia. Curr Biol. 2007;17(1):79–84. doi: 10.1016/j.cub.2006.11.022 17208190 10.1016/j.cub.2006.11.022

[65] Jasnin M, Asano S, Gouin E, Hegerl R, Plitzko JM, Villa E, et al Three-dimensional architecture of actin filaments in Listeria monocytogenes comet tails. Proceedings of the National Academy of Sciences of the United States of America. 2013;110:20521–20526. doi: 10.1073/pnas.1320155110 24306931 10.1073/pnas.1320155110 PMC3870744

[66] Korobova F, Svitkina T. Arp2/3 complex is important for filopodia formation, growth cone motility, and neuritogenesis in neuronal cells. Mol Biol Cell. 2008;19:1561–1574. doi: 10.1091/mbc.E07-09-0964 18256280 10.1091/mbc.E07-09-0964 PMC2291425

[67] Gallo G. The cytoskeletal and signaling mechanisms of axon collateral branching. Developmental neurobiology. 2011;71:201–220. doi: 10.1002/dneu.20852 21308993 10.1002/dneu.20852

[68] Ishikawa R, Sakamoto T, Ando T, Higashi-Fujime S, Kohama K. Polarized actin bundles formed by human fascin-1: their sliding and disassembly on myosin II and myosin V in vitro. J Neurochem. 2003;87:676–685. doi: 10.1046/j.1471-4159.2003.02058.x 14535950 10.1046/j.1471-4159.2003.02058.x

[69] Volkmann N, DeRosier D, Matsudaira P, Hanein D. An atomic model of actin filaments cross-linked by fimbrin and its implications for bundle assembly and function. J Cell Biol. 2001;153:947–956. doi: 10.1083/jcb.153.5.947 11381081 10.1083/jcb.153.5.947 PMC2174342

[70] Shibata Y, Voeltz GK, Rapoport TA. Rough sheets and smooth tubules. Cell. 2006;126:435–439. doi: 10.1016/j.cell.2006.07.019 16901774 10.1016/j.cell.2006.07.019

[71] Korkhov VM, Zuber B. Direct observation of molecular arrays in the organized smooth endoplasmic reticulum. BMC cell biology. 2009;10:59 doi: 10.1186/1471-2121-10-59 19703297 10.1186/1471-2121-10-59 PMC2737311

[72] Terasaki M, Chen LB, Fujiwara K. Microtubules and the endoplasmic reticulum are highly interdependent structures. J Cell Biol. 1986;103:1557–1568. doi: 10.1083/jcb.103.4.1557 3533956 10.1083/jcb.103.4.1557 PMC2114338

[73] Klumperman J, Raposo G. The complex ultrastructure of the endolysosomal system. Cold Spring Harbor perspectives in biology. 2014;6:a016857 doi: 10.1101/cshperspect.a016857 24851870 10.1101/cshperspect.a016857 PMC4176003

[74] Murk JL, Humbel BM, Ziese U, Griffith JM, Posthuma G, Slot JW, et al Endosomal compartmentalization in three dimensions: implications for membrane fusion. Proc Natl Acad Sci U S A. 2003;100(23):13332–7. doi: 10.1073/pnas.2232379100 14597718 10.1073/pnas.2232379100 PMC263806

[75] Vaughn JE. Fine structure of synaptogenesis in the vertebrate central nervous system. Synapse. 1989;3(3):255–85. doi: 10.1002/syn.890030312 2655146 10.1002/syn.890030312

[76] Gundelfinger ED, Reissner C, Garner CC. Role of Bassoon and Piccolo in Assembly and Molecular Organization of the Active Zone. Front Synaptic Neurosci. 2015;7:19 doi: 10.3389/fnsyn.2015.00019 26793095 10.3389/fnsyn.2015.00019 PMC4709825

[77] Zhang Z, Wu Y, Wang Z, Dunning FM, Rehfuss J, Ramanan D, et al Release mode of large and small dense-core vesicles specified by different synaptotagmin isoforms in PC12 cells. Mol Biol Cell. 2011;22(13):2324–36. doi: 10.1091/mbc.E11-02-0159 21551071 10.1091/mbc.E11-02-0159 PMC3128534

[78] van den Pol AN. Neuropeptide transmission in brain circuits. Neuron. 2012;76(1):98–115. doi: 10.1016/j.neuron.2012.09.014 23040809 10.1016/j.neuron.2012.09.014 PMC3918222

[79] Bourne JN, Harris KM. Coordination of size and number of excitatory and inhibitory synapses results in a balanced structural plasticity along mature hippocampal CA1 dendrites during LTP. Hippocampus. 2011;21:354–373. doi: 10.1002/hipo.20768 20101601 10.1002/hipo.20768 PMC2891364

[80] Fernández-Busnadiego R, Asano S, Oprisoreanu AM, Sakata E, Doengi M, Kochovski Z, et al Cryo-electron tomography reveals a critical role of RIM1*α* in synaptic vesicle tethering. J Cell Biol. 2013;201(5):725–740. doi: 10.1083/jcb.201206063 23712261 10.1083/jcb.201206063 PMC3664715

[81] Vargas KJ, Schrod N, Davis T, Fernandez-Busnadiego R, Taguchi YV, Laugks U, et al Synucleins Have Multiple Effects on Presynaptic Architecture. Cell reports. 2017;18:161–173. doi: 10.1016/j.celrep.2016.12.023 28052246 10.1016/j.celrep.2016.12.023 PMC5510332

[82] Marko M, Hsieh C, Schalek R, Frank J, Mannella C. Focused-ion-beam thinning of frozen-hydrated biological specimens for cryo-electron microscopy. Nat Methods. 2007;4(3):215–217. doi: 10.1038/nmeth1014 17277781 10.1038/nmeth1014

[83] Rigort A, Bäuerlein FJB, Villa E, Eibauer M, Laugks T, Baumeister W, et al Focused ion beam micromachining of eukaryotic cells for cryoelectron tomography. Proc Natl Acad Sci U S A. 2012;109(12):4449–4454. doi: 10.1073/pnas.1201333109 22392984 10.1073/pnas.1201333109 PMC3311327

[84] Fernandez JJ, Laugks U, Schaffer M, Bäuerlein FJB, Khoshouei M, Baumeister W, et al Removing Contamination-Induced Reconstruction Artifacts from Cryo-electron Tomograms. Biophys J. 2016;110(4):850–859. doi: 10.1016/j.bpj.2015.10.043 26743046 10.1016/j.bpj.2015.10.043 PMC4775855

[85] Renvoisé B, Blackstone C. Emerging themes of ER organization in the development and maintenance of axons. Curr Opin Neurobiol. 2010;20:531–537. doi: 10.1016/j.conb.2010.07.001 20678923 10.1016/j.conb.2010.07.001 PMC2946456

[86] Spacek J, Harris KM. Three-dimensional organization of smooth endoplasmic reticulum in hippocampal CA1 dendrites and dendritic spines of the immature and mature rat. J Neurosci. 1997;17:190–203. doi: 10.1523/JNEUROSCI.17-01-00190.1997 8987748 10.1523/JNEUROSCI.17-01-00190.1997 PMC6793680

[87] Phillips MJ, Voeltz GK. Structure and function of ER membrane contact sites with other organelles. Nat Rev Mol Cell Biol. 2016;17:69–82. doi: 10.1038/nrm.2015.8 26627931 10.1038/nrm.2015.8 PMC5117888

[88] Pickel VM, Chan J, Veznedaroglu E, Milner TA. Neuropeptide Y and dynorphin-immunoreactive large dense-core vesicles are strategically localized for presynaptic modulation in the hippocampal formation and substantia nigra. Synapse. 1995;19(3):160–169. doi: 10.1002/syn.890190303 7784956 10.1002/syn.890190303

[89] Takamori S, Holt M, Stenius K, Lemke EA, Gronborg M, Riedel D, et al Molecular anatomy of a trafficking organelle. Cell. 2006;127(4):831–46. doi: 10.1016/j.cell.2006.10.030 17110340 10.1016/j.cell.2006.10.030

[90] Sabo SL, Gomes RA, McAllister AK. Formation of presynaptic terminals at predefined sites along axons. J Neurosci. 2006;26:10813–10825. doi: 10.1523/JNEUROSCI.2052-06.2006 17050720 10.1523/JNEUROSCI.2052-06.2006 PMC6674732

[91] Verderio C, Coco S, Bacci A, Rossetto O, De Camilli P, Montecucco C, et al Tetanus toxin blocks the exocytosis of synaptic vesicles clustered at synapses but not of synaptic vesicles in isolated axons. Journal of Neuroscience. 1999;19(16):6723–6732. doi: 10.1523/JNEUROSCI.19-16-06723.1999 10436029 10.1523/JNEUROSCI.19-16-06723.1999 PMC6782867

